# Rubber Elongation Factor (REF), a Major Allergen Component in *Hevea brasiliensis* Latex Has Amyloid Properties

**DOI:** 10.1371/journal.pone.0048065

**Published:** 2012-10-25

**Authors:** Karine Berthelot, Sophie Lecomte, Yannick Estevez, Bénédicte Coulary-Salin, Ahmed Bentaleb, Christophe Cullin, Alain Deffieux, Frédéric Peruch

**Affiliations:** 1 CNRS, LCPO, UMR 5629, Pessac, France; 2 CNRS, CBMN, UMR 5248, Pessac, France; 3 Univ. Bordeaux 2, CNRS, IBGC, UMR 5095, Bordeaux, France; 4 CNRS, CRPP, UPR 8641, Pessac, France; University of Crete, Greece

## Abstract

REF (Hevb1) and SRPP (Hevb3) are two major components of *Hevea brasiliensis* latex, well known for their allergenic properties. They are obviously taking part in the biosynthesis of natural rubber, but their exact function is still unclear. They could be involved in defense/stress mechanisms after tapping or directly acting on the isoprenoid biosynthetic pathway. The structure of these two proteins is still not described. In this work, it was discovered that REF has amyloid properties, contrary to SRPP. We investigated their structure by CD, TEM, ATR-FTIR and WAXS and neatly showed the presence of β-sheet organized aggregates for REF, whereas SRPP mainly fold as a helical protein. Both proteins are highly hydrophobic but differ in their interaction with lipid monolayers used to mimic the monomembrane surrounding the rubber particles. Ellipsometry experiments showed that REF seems to penetrate deeply into the monolayer and SRPP only binds to the lipid surface. These results could therefore clarify the role of these two paralogous proteins in latex production, either in the coagulation of natural rubber or in stress-related responses. To our knowledge, this is the first report of an amyloid formed from a plant protein. This suggests also the presence of functional amyloid in the plant kingdom.

## Introduction

*Hevea brasiliensis* (Willd. Ex A. Juss) Müll. Arg (rubber tree) is a tropical plant belonging to the *Euphorbiaceae* family, which is cultivated worldwide and especially in Southeast Asia, to produce natural rubber (NR). NR is a *cis*-1,4-polyisoprene biopolymer of high economic importance (for review see [1]), which is produced from the latex after tapping. Latex is a complex white cytoplasmic system produced by plant laticifers, which contains mainly rubber and non-rubber particles, organelles, proteins and cytoplasmic C-serum [2]. The hevea latex contains many proteins and at least 13 are allergens, as Hevb3 and Hevb1 [3], [4], [5], [6], [7]. Both proteins are abundant and associated with latex allergy, particularly in individuals with *Spina bifida* who undergo frequent surgeries [8], [9], [10]. REF and SRPP share several IgE epitopes [11], but their role in the immune mechanism leading to latex hypersensitivity has not yet been determined. They are also named Small Rubber Particle Protein (SRPP) and the Rubber Elongation Factor (REF) [8], [12], [13]. SRPP is specifically localized in the laticifer layers in the conducting phloem, whereas REF is localized in all laticifer layers [14]. Indeed, REF and SRPP have been respectively visualized by immunogold electron microscopy on the Large Rubber Particles (LRP, generally above 0.4 µM in diameter) and the Small Rubber Particles (SRP, smaller than 0.4 µM in diameter) [8], [12], [15], [16], [17]. In addition, SRPs were also described as having much higher enzymatic activity at their surface than LRPs do [16], [18].

The NR synthesis is regulated by the activity of rubber particle-associated proteins present at the surface of the membrane monolayer, which surrounds the rubber particles [19], [20], [21]. Various enzymes such as prenyl transferases or synthases have been characterized in latex and are clearly elongating the rubber polymer by condensing isopentenyldiphosphate (IPP) with dimethylallyldiphosphate (DMAPP) or isoprenyl intermediates [22], [23]. Three more proteins, SRPP, the Guayule Homologue of SRPP (GHS) and REF were also described as having a positive effect on rubber elongation [12], [13], [24], [25]. *Hevea* genes encoding for REF and SRPP proteins have been cloned [5], [13], [26], [27] and various isoforms identified [28], [29]. Messenger RNAs of REF and SRPP have been found to be highly expressed in the *Hevea* latex and laticifers [28], [30], [31], [32]. Tree tapping stimulates the gene expression of both proteins [32], [33]. REF expression is also positively correlated with latex yield in *Hevea* [32] and Dennis and Light [12] also showed that the amount of REF protein in the whole latex was proportional to rubber content. REF and SRPP proteins from hevea latex are acidic proteins respectively of 14.6 and 24 kDa, which were believed to not be post-translationally modified [8]. But recently, SRPP was described as a glycoprotein susceptible to interact with a hevea latex lectin present on lutoids and susceptible to play a role in latex coagulation [25]. REF was also shown as a native protein able to tetramerize as a protein of about 58 kDa [4]. However, no information is available on their respective structure.

In order to evaluate the possible functions of REF and SRPP in latex, we purified in this study both recombinant proteins and analyzed various biochemical and structural properties. As REF displayed aggregative and amyloid features, we characterized its structure by circular dichroism (CD), transmission electronic microscopy (TEM), infra-red spectroscopy (ATR-FTIR) and X-ray diffraction (WAXS). To go further in the comparison of REF and SRPP function, we also evaluated the kind of interaction that each protein could have with various lipids, using dot blots (bulk lipids) or model lipid monolayers at the air-water interface. If both proteins are really closely related in term of amino-acid sequences, we demonstrate in this study that their structure are neatly different (REF being able to switch into an amyloid form), and that their mode of interaction with the surface of the lipid monolayer (mimicking the one surrounding latex particle) is also different, which may suppose also different roles of REF and SRPP, particularly in natural rubber biosynthesis.

**Table 1:** ATR-FTIR analyses of REF and SRPP.

Secondary structure element	*Amide I wavenumbers (cm^−1^)*	*Relative comparative (%)*	
	REF, SRPP	REF	SRPP
**Aggregation**	1614, 1614	9.12	6.52
**β-sheet**	1630, 1629	37.81	20.39
**Random coil**	1646, 1642	15.20	24.59
**Helix**	1657, 1656	14.25	21.04
**Turns**	1670, 1670	11.37	13.61
**Hydrophobic domain**	1685, 1685	12.25	13.85

**Figure 1:**
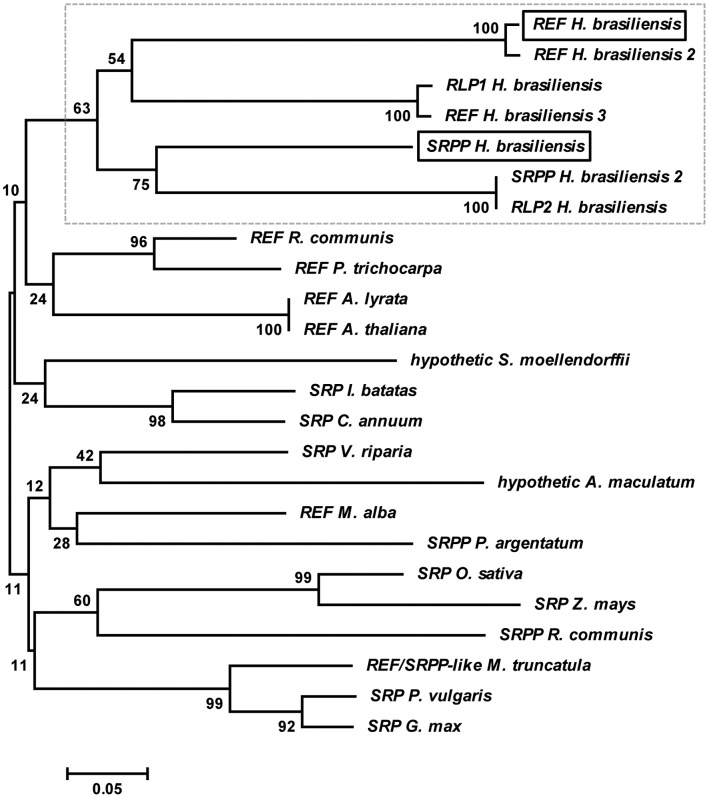
Phylogenetic analysis of REF and SRPP protein family. A BlastP was realized on REF P15252 at http://www.phylogeny.fr. The accession numbers of each 24 aligned sequences with ClustalW (http://www.genome.jp/tools/clustalw/program) were as follows: REF *Hevea brasiliensis* P15252, REF *Hevea brasiliensis* AEH05970 (2), REF *Hevea brasiliensis* AAR11448 (3), SRPP *Hevea brasiliensis* O82803, SRPP *Hevea brasiliensis* AAO66432 (2), RLP1 *Hevea brasiliensis* AAP46159, RLP2 *Hevea brasiliensis* AAP46160, REF *Ricinus communis* XP_002512427, REF *Arabidopsis thaliana* NP_187201, SRP *Vitis riparia* Q9SW70, REF *Morus alba* ACV90044, REF *Amblyomma maculatum* AEO33677, SRP *Ipomoea batatas* ABP35522, SRP *Oryza sativa* AAO72547, SRP *Zea mays* ACG39345, REF *Selaginella moellendorffii* XP_002969776, SRPP *Parthenium argentatum* AAQ11374, SRP *Capsicum annuum* ADI60300, SRPP *Ricinus communis* XP_002514917, REF *Populus trichocarpa* XP_002319520, REF *Arabidopsis lyrata* XP_002882419, SRP *Glycine max* XP_003543052, REF/SRPP-like protein *Medicago truncatula* XP_003593563. Evolutionary analyses were conducted in MEGA5 [65] using the Neighbor-Joining method, the bootstrap test (1000 replicates) and the p-distance method. All positions containing gaps and missing data were eliminated. On the right, characterized proteins are presented. SRPP: Small Rubber Particle Protein; REF: Rubber Elongation Factor; SRP: Stress-related protein; RLP: REF-like stress related protein. Both REF and SRPP proteins used in this study are framed. *Hevea brasiliensis* protein family is framed by a gray dashed box.

## Materials and Methods

### REF and SRPP Cloning, Expression and Purification

The SRPP gene (GenBank accession no. AJ223388) and the REF gene (GenBank accession no. X56535) were synthesized by GenScript (Piscataway, USA) after codon optimization and addition of a 6-histidine tag at the N-ter. Then they were cloned, respectively, in the *NdeI–XhoI* site of pET24a (Novagen Inc., WI, USA) to construct pET24a-6His-SRPP and pET24a-6His-REF. Each plasmid was introduced in *Escherichia coli* BL21 (DE3) pLysS Gold cells. Bacteria were grown to 0.7 OD in 2×YT medium (16 g/L tryptone, 10 g/L yeast extract, and 5.0 g/L NaCl), and expression was induced by addition of 1 mM isopropyl-D-thiogalactoside (Euromedex, Souffelweyersheim, France). After 4 h induction, cells were harvested by centrifugation and frozen at −20°C. Overexpression of SRPP and REF caused inclusion body formation. Cells were sonicated 5×1 min in buffer A (150 mM NaCl and 100 mM Tris-HCl, pH 8.0). The lysate was centrifuged for 30 min at 20,000 *g*. The pellet was washed in the buffer A and resuspended in denaturing buffer (8 M urea in buffer A). The lysate was incubated with 2 mL Ni-NTA resin (InVitrogen, ThermoScientifique, Illkirch, France) for 1 h at room temperature. The resin was then washed twice with 35 mL of 8 M urea/buffer A, by centrifuging 10 min at 900 *g*. The proteins were eluted from the resin in the same buffer containing 250 mM imidazole (Euromedex). Protein samples were pooled and dialyzed against buffer A ±10% glycerol and kept aliquoted at −80°C. This yields ∼2–4 mg of peptide per liter of culture. The peptide was pure as judged by analysis on 15% sodium dodecyl sulfate polyacrylamide gel electrophoresis followed by Coomassie Blue staining. Protein concentrations were determined by quantitative amino-acid analysis.

**Figure 2:**
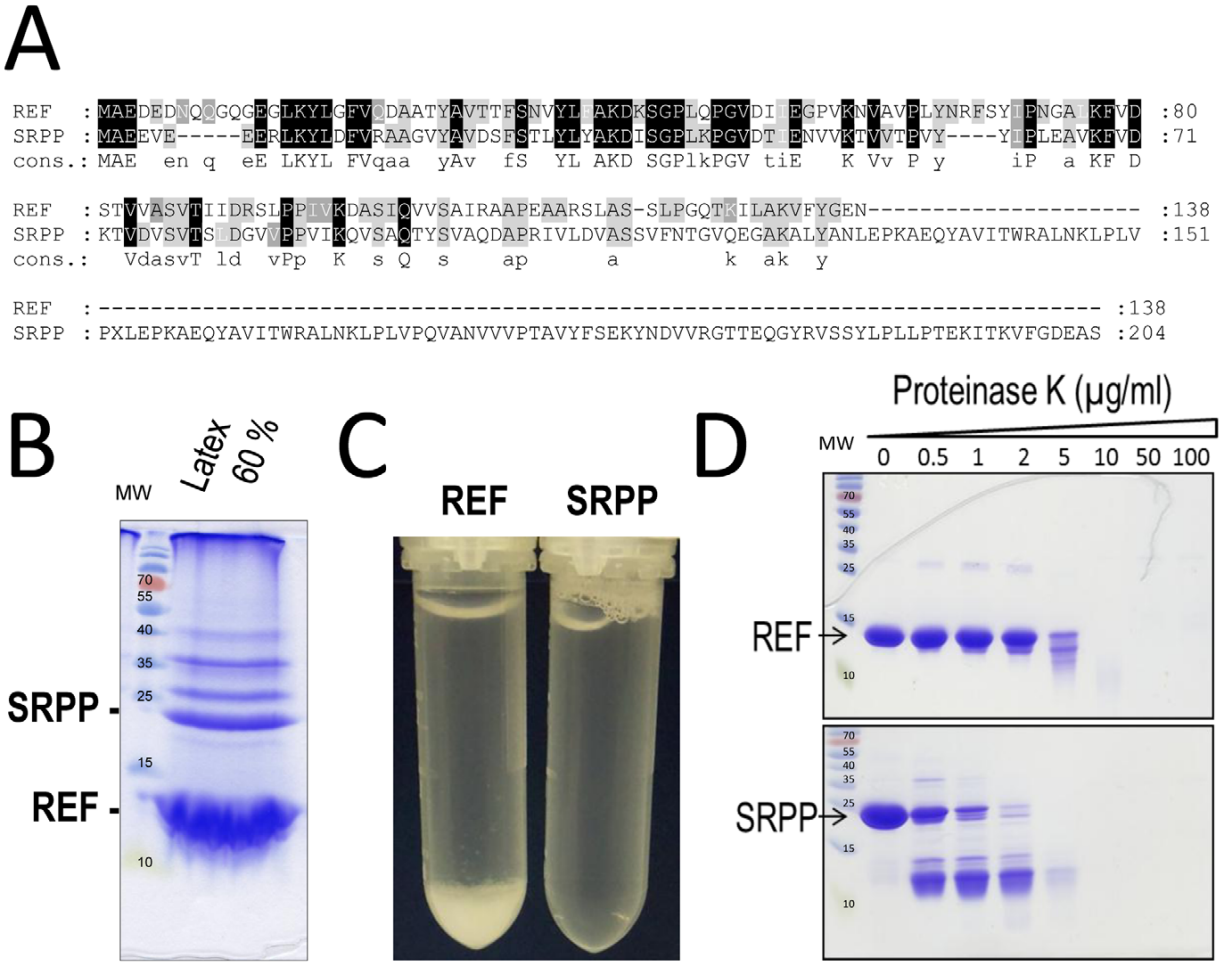
REF and SRPP are major latex proteins with homology but distinct aggregation properties. A. Sequence homology of REF and SRPP after alignment with ClustalW (http://www.genome.jp/tools/clustalw/program) and shading by using GeneDoc 2.7.0 (http://www.nrbsc.org/downloads/). Cons: consensus sequence for homology in proteins found in *Hevea brasiliensis* and showed in Supplementary Figure S1. **B**. Protein content of 60% latex by 15% SDS-PAGE. **C.** Aggregation state of REF and SRPP at 50 µM in PBS buffer 1 X after 3 h at 37°C. **D.** Differential Proteinase K resistance of REF and SRPP after incubation with Proteinase K, 30 min at 37°C and visualization on 15% SDS-PAGE.

For polymerization experiments, proteins were usually incubated at 20 µM in 1× PBS buffer pH 7.4 (Euromedex) at 37° for 3 h, and kept at 4°C.

**Figure 3:**
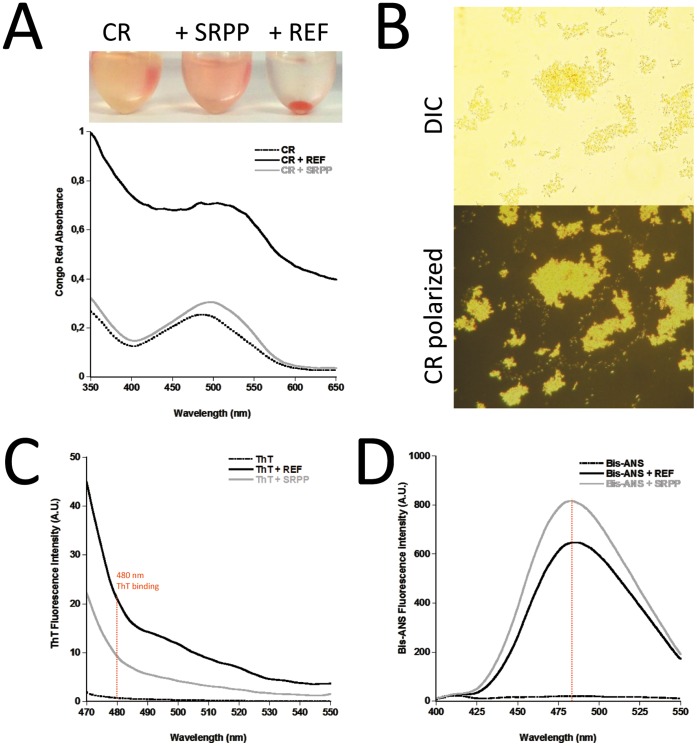
Amyloid dye-binding properties of REF and SRPP. **A.** Samples (top image) and absorbance spectra in presence of Congo red (CR). **B.** Birefringence of REF red aggregates under visible light (top) and cross-polarized light (bottom). **C.** ThT binding spectra. **D.** Bis-ANS binding spectra.

### Limited Proteolysis with Proteinase K

Proteins were incubated at 100 µM in 1× PBS for 3 h at 37°C. Height µl of each 100 µM protein solution were mixed with 8 µl of an appropriate dilution of proteinase K (Euromedex), and incubated at 37°C for 30 min. Digestion was stopped by adding electrophoresis sample buffer pH 6.8, containing 4% SDS, 2% mercaptoethanol (v/v), 12% glycerol (w/v), 0.01% Serva Blue G, and phenylmethylsulfonyl fluoride (1 mM). The mixture was then immediately heated at 100°C for 5 min. Samples were analyzed by 15% SDS-PAGE and stained with Coomassie blue.

### ThT, Bis-ANS, Congo Red Binding and Birefringence

Data were obtained with a POLARstar Omega microplate reader (BMG Labtech, Champigny sur- Marne, France) and a Perkin-Elmer LS50 fluorescence spectrometer. For ThT binding, ThT (Sigma, St. Louis, MO, USA) was used at a concentration of 10 µM with λ_excitation_ at 440/450 nm and λ_emission_ at 480 nm. For Bis-ANS binding, the dye stock (3 mM) was used at 1 µM final concentration with λ_excitation_ at 390 nm and λ_emission_ at 540 nm. A 20-µM solution of CR (C.I. 22120; Sigma) in PBS, pH 7.4, was prepared (extinction coefficient at 498 nm, ∼3.7×10^4^ M^−1^.cm^−1^) and filtered through a 0.22-µm polyether sulfone filter. Absorbance spectra with CR (5 µL for 100 µL proteins at 20 µM incubated 30 min) were extracted with the MARS Data Analysis Software 2.10 R3 (BMG Labtech). For microscopic observations stained aggregates were centrifuged at 16,000 *g* for 1 min and spread dried on microscope slides. CR binding and birefringence were examined with an ECLIPSE E600FN microscope (Nikon Instruments Europe, Amstelveen, The Netherlands) with a PlanFluor 40× objective and a DXM1200 digital camera (Nikon Instruments Europe) equipped with optimally aligned cross-polarizers.

**Figure 4:**
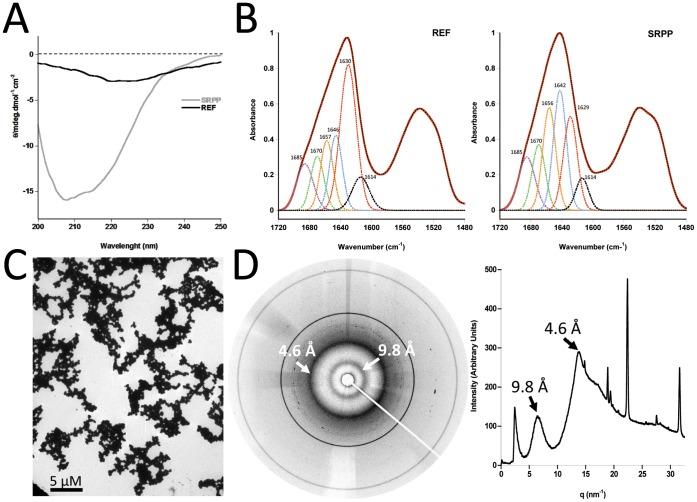
Amyloid structural properties of REF aggregates. **A.** Circular dichroism spectra of REF aggregated protein compared to soluble SRPP protein. **B.** ATR-FTIR spectra of air-dried aggregated REF and soluble SRPP. Deconvolutions of the amide I band are presented for each protein. **C.** TEM image of REF amorphous aggregates obtained at the end of the polymerization. **D.** Analysis by WAXS of REF lyophilized aggregates. The diffraction rays at about 4.7 Å/10 Å, characteristic of the amyloid cross-beta core are showed by the arrows. Image has a 345 mm diameter corresponding to 2300 p×2300 p (0.15 mm/p). In these experiments aggregates were grown 3 h at 37°C and 20 µM and kept at 4°C.

### Circular Dichroism (CD) Spectroscopy

Far-UV CD spectra were collected at 20°C, using a Jasco JS 810 spectropolarimeter (Jasco, Tokyo, Japan) equipped with a JASCO PTC-423 S/15 temperature controller. Measurements were done with a quartz cell with a 0.1 cm path length (Hellma-France). Protein concentration was 10 µM in PBS buffer.

### ATR–FTIR Spectroscopy

REF aggregates were washed twice in water by centrifugation (16,000 *g*, 10 min, 4°C) to remove glycerol. SRPP soluble proteins were dialyzed overnight at 4°C in PBS 1× using Slide-A-Lyser 3.5 K dialysis cassettes (Pierce, Thermo Fisher Scientific, Rockford, IL, USA). Five microliters of each sample was deposited on a germanium ATR crystal (Specac, Orpington, UK) and left to evaporate in room air. ATR–FTIR spectra were recorded on a Nicolet Nexus 870 FTIR spectrometer equipped with a mercury cadmium telluride detector (Thermo Fisher Scientific, San Jose, CA, USA), with a spectral resolution of 4 cm^−1^ and a one-level zero filling. Two hundred interferograms, representing an acquisition time of 7 min, were co-added.

The resulting spectra were analyzed with an algorithm based on a second-derivative function and a self-deconvolution procedure (GRAMS and OMNIC softwares, ThermoFisher Scientific) to determine the number and wavenumber of individual bands within the spectral range 1485–1750 cm^−1^. The amide I band of each spectrum could be fitted by six bands assigned to the vibration of amide I involved in six different secondary structures. Vibrational assignments of the infrared band components in the amide I region were made according to Goormaghtigh et al.1994 [34]. The fit was obtained with a mixed Lorentzian (70%)–Gaussian (30%) band profile and width at half-height included between 15% and 45%. The relative contributions of the different bands were determined from the fit results obtained for the amide I band. The amount of each secondary-structure element is given as a percentage (Table 1) and is determined by dividing the integral intensity of one amide I band component by the total intensity of all amide I band components. The standard error does not exceed 1.5%. For all spectra, fit results correspond to a local minimum at the end of the iteration procedure.

**Figure 5:**
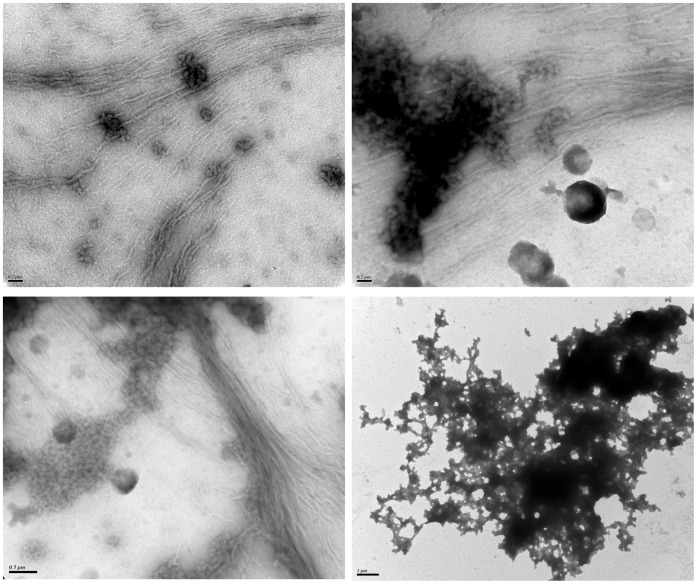
REF self-assembles rapidly as amyloid fibers and amorphous aggregates. In order to visualize early step of fibrillization, we incubated the sample at 20°C, 20 µM and 20 min. The sample was composed of large (about 10 nm) and µM-long fibers assembling laterally and a lot of amorphous aggregates.

### Transmission Electron Microscopy (TEM)

REF proteins after incubation in PBS were absorbed 10 min onto Formvar-coated, carbon-stabilized copper grids (400 mesh) and washed 3 times with water. Grids were then negatively stained 1 min with 10 µL of freshly prepared 2% uranyl acetate in water, dried with filter paper, and examined with a Hitachi H7650 transmission electron microscope (Hitachi, Krefeld, Germany) at an accelerating voltage of 80 kV. TEM was performed at the Pôle Imagerie Electronique of the Bordeaux Imaging Center.

**Figure 6:**
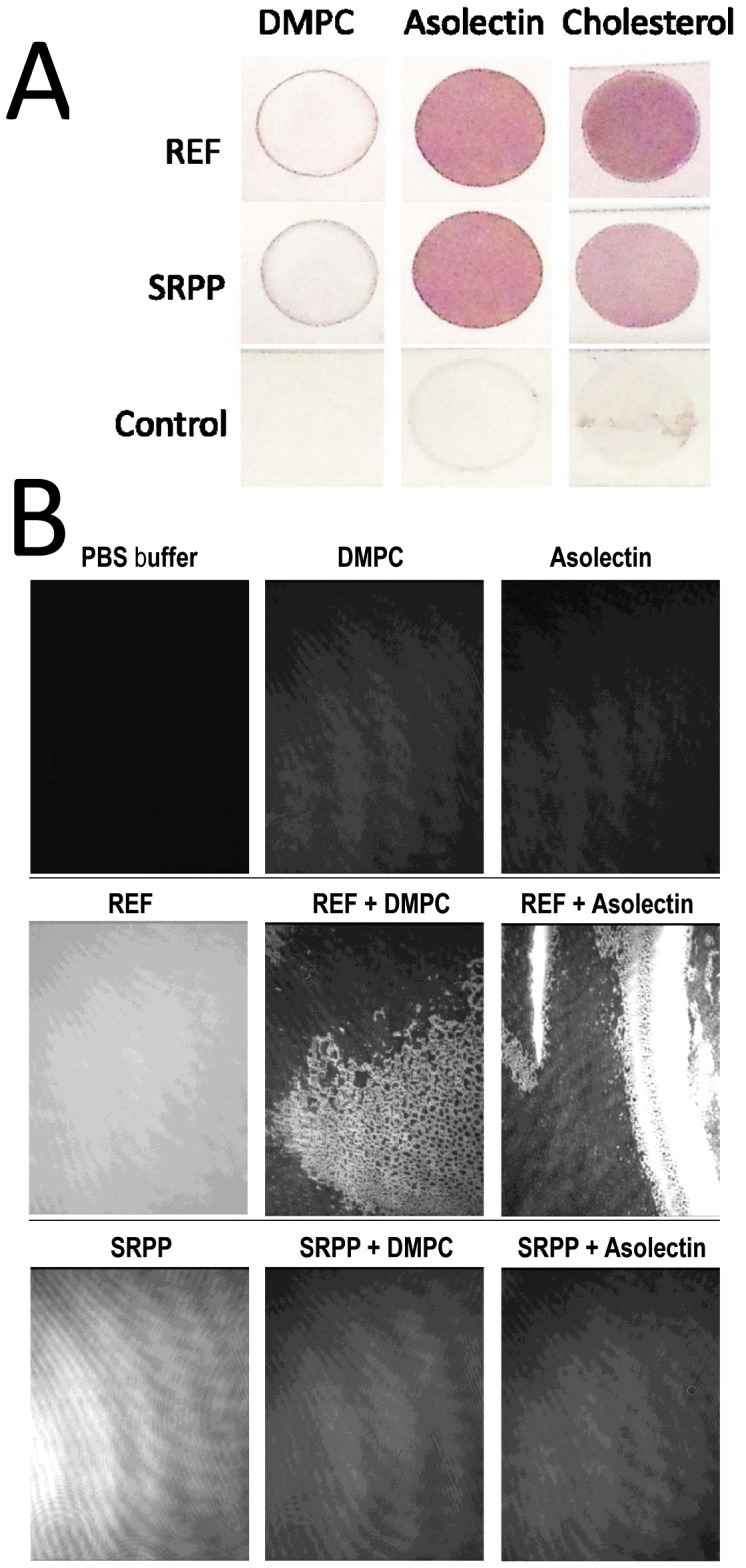
REF and SRPP interact differently with membrane lipids. **A.** Dot blots of DMPC, asolectin, cholesterol have been incubated overnight at 20°C with 20 µM of each protein. Proteins interacting with lipids were revealed with mouse anti-histidine antibody and then anti-mouse-phosphatase-alkaline antibody followed by a NBT/BCIP staining. **B.** Ellipsometric images of REF and SRPP interacting with DMPC and asolectin monolayers at the air/water or lipid interface.

**Table 2:** Thickness of the surface layers and ellipsometric angles before and after interaction of REF and SRPP with PBS pH 7.4 as subphase.

	*Ellipsometric angles (δΔ, Ψ) and layer thickness* c					
	*Protein at air/water interface*		*Lipid monolayer + protein* a			
			*Asolectin*		*DMPC*	
	|δΔ|°^b^	Thickness	|δΔ|°	Thickness	|δΔ|°	Thickness
	Ψ°	Å	Ψ°	Å	Ψ°	Å
**No protein**	N/A	N/A	6.36±0.15	17.80±0.50	4.57±0.37	**16.30±1.32**
	N/A		2.40±0.12		2.40±0.12	
**REF**	38.12±1.80	**136.1±6.40**	18.72±1.69	66.85±6.00	17.84±0.84	**63.71±3.00**
	2.40±0.12		2.40±0.12		2.40±0.12	
**SRPP**	10.15±0.12	**36.25±0.40**	7.52±0.52	26.85±2.00	7.01±0.52	**25.10±1.85**
	2.40±0.12		2.40±0.12		2.40±0.12	

N/A, not applicable.

a Lipid monolayers were formed at a constant pressure of 28 mN/m and at 25°C. REF and SRPP were injected at 1 µM in a PBS 1X buffer subphase.

b Ellipsometry angles |δΔ| and Ψ; |δΔ| = |Δ−Δ_0_|. Pressure was at the equilibrium state (less than 1 h), when the pressure values were at plateau.

c The thickness was determined using a mean value of 1.45 for the refractive index. Results are presented as mean ± SD. Corresponding ellipsometric images are presented in Figure 6b.

### Wide-Angle X-ray Scattering (WAXS)

REF aggregates were washed in water and lyophilized with a CHRIST Freeze-drier (Osterode am Harz, Germany). The powder was introduced into 1 mm diameter glass capillaries (Glaskapillaren GLAS, Glas-Technik & Konstruktion, Schönwalde-Glien, Germany). X-ray data were collected at the Centre de Recherche Paul Pascal (CRPP, Pessac, France) on a Rigaku Nanoviewer (XRF microsource generator, MicroMax 007HF), with an 1200-W rotating anode coupled to a confocal Max-Flux® Osmic mirror (Applied Rigaku Technologies, Austin, USA) and a MAR345 image plate detector (MARResearch, Norderstedt, Germany). Spectra were integrated with the FIT2D software (ERSF; http://www.esrf.eu/). A wide-angle X-ray scattering with transmission geometry setting was chosen, with a sample-detector distance of 153.6 mm and a 3600 s accumulation, providing access to periodicities in the 35–2.5 Å range. Data were processed by subtracting the scattering pattern due to the air.

**Table 3:** Surface pressures of REF and SRPP proteins at the air-water interface or in presence of lipid monolayers.

Proteins	Pressure air/water interface^a^	ΔP (mN/m) with lipid monolayers^b^	
	(mN/m)	Asolectin	DMPC
**REF**	24.6	4.5	10.4
**SRPP**	28.3	5.6	5.3

a REF and SRPP were injected at 1 µM in the PBS 1X buffer subphase and pressure was measured when values were at plateau, usually after less than 1 h.

b Lipid monolayers were formed at a constant pressure of 28 mN/m and at 25°C. REF and SRPP were then injected at 1 µM in the PBS 1X buffer subphase. ΔP corresponds at the variation of pressure observed after injection of the proteins under the lipid monolayer and the complete stabilization of the interface.

**Figure 7:**
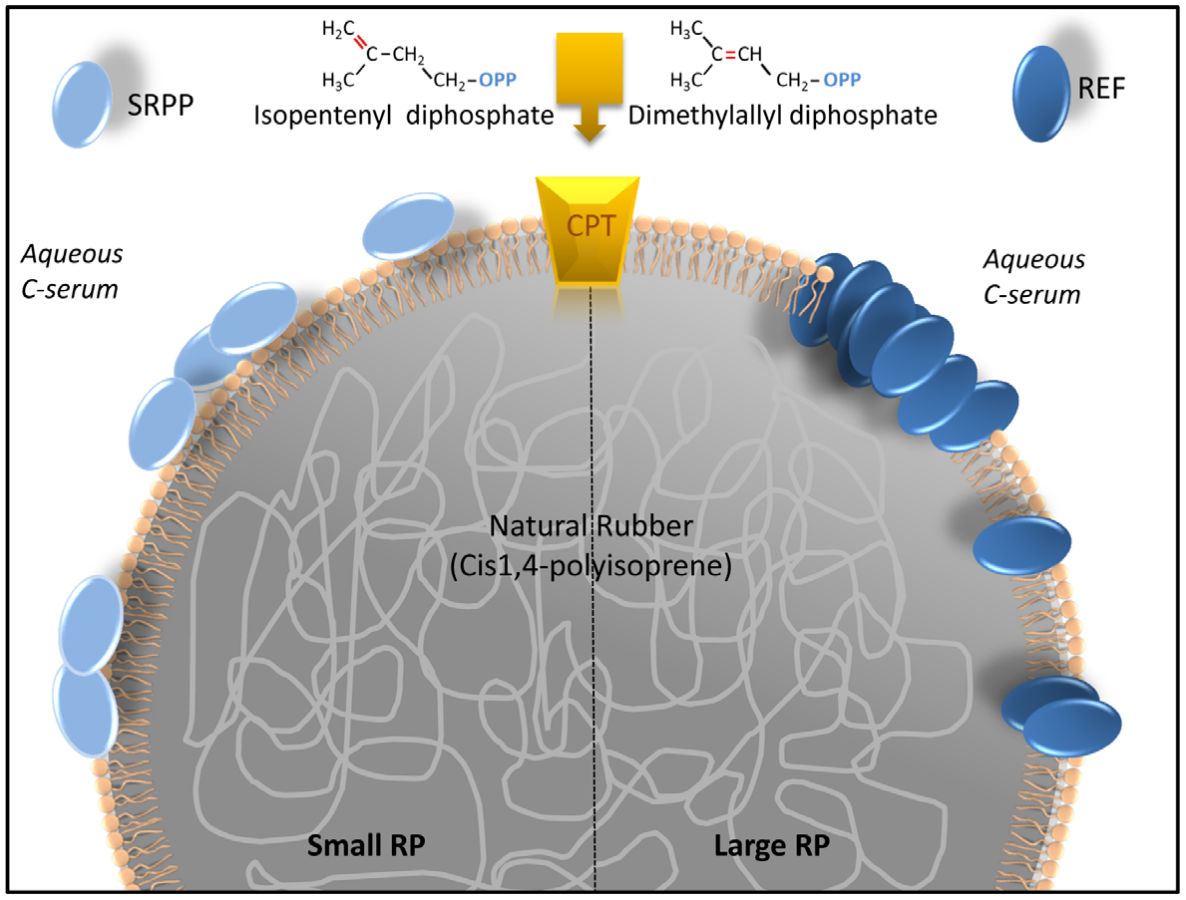
Proposed model of REF and SRPP interaction with the monolayer membrane of the rubber particle. SRPP is bound to the surface of the Small Rubber Particle (SRP) while REF is inserted into the membrane of the Large Rubber Particle (LRP). Other proteins are also transmembrane proteins such as cis-prenyl transferases (CPT) or rubber transferases able to condensate and elongate natural rubber from isopentenyl and dimethylallyl diphosphates.

### Protein/lipid Interactions on Dot Blots

Lipid dot blots were performed as previously described with few modifications [35]. Indeed, 4 µL of DMPC at 2 mg/mL (1,2-dimyristoyl-sn-glycero-3-phosphocholine; Aventi Polar Lipids, Inc., Alabaster, AL USA) or cholesterol or asolectin (Sigma) were allowed to dry on a PVDF transfer membrane (Hybond™-P, GE Healthcare Europe GmbH, Orsay, France) for 5 min. The lipid blotted membranes were then incubated at room temperature overnight with 2 mL of 20 µM WT or M8 proteins. Blots were washed twice with 1X PBS and blocked with PBS 5% screamed dried milk (Régilait, Saint Martin Belle Roche France). To detect the proteins bound to blotted DMPC, asolectin or cholesterol, mouse anti-His-tag antibody (GE Healthcare), anti-mouse-phosphatase-alkaline antibody (Sigma) and NBT/BCIP (Euromedex) were used.

### Ellipsometry Images and Measurements

Adsorption experiments were performed at 25±1°C on a circular Teflon trough (20.4 cm^2^). The surface pressure (π) was measured with a plate of Whatman filter paper held by a Nima Wilhelmy balance. The trough was filled with 8 ml of 1× PBS buffer (pH 7.4). The surface tension of the water was measured at 72.8 mN.m^−1^. The interaction of proteins with lipid films (asolectin or DMPC) was performed in two steps. First, a homogeneous Langmuir monolayer of lipids was formed by spreading a 1 mg/mL solution (in 20% methanol/80% chloroforme) at the air/water interface to finally reach a constant surface pressure of 28 mN.m^−1^. Second, freshly defrozen and non-glycerolated proteins were injected at a concentration of 1 µM into the subphase. The surface pressure was continuously monitored. Images were obtained with an NFT IElli2000 ellipsometer (NFT, Göttingen, Germany) equipped with a double-frequency Nd–Yag laser (532 nm, 50 mW), a polarizer, an analyzer, and a CCD camera. The film morphology at the interface was observed by the CCD camera. An incidence angle of 54.58°, which gives the null conditions, was used. All images were corrected from the tilt angle observation. The spatial resolution was about 2 µm, and the image size was 450×670 µm, with a 10× magnification lens. Thickness was determined using a mean value of 1.45 for the refractive index.

## Results

### REF and SRPP are Mainly Plant Proteins from the Same Family

We performed a BLAST on the REF amino-acid sequence (GeneBank accession number P15252), and aligned the sequences of 24 predicted proteins with ClustalW. We then realized a phylogenetic analysis (Figure 1 and Supplementary Figure S1). The results shows quite related proteins, mainly from plant origins. HbREF (Hevb1), HbSRPP (Hevb3) from hevea, and *Parthenium argentatum* GHS are the only proteins from latex-producing plants and several other proteins are stress-related proteins (SRP). The proteins from *Hevea brasiliensis* obviously all originate from a common ancestor. From our analysis a lot of genes are predicted to produce short proteins, which are ranging from 117 to 298 amino acids. HbREF, HbSRPP and PaSRPP (GHS) are the only well characterized proteins, and they are all present on rubber particles but with different molecular weights (respectively 14.5, 24, and 26.2 kDa). Recently another homologous stress-related gene was identified in *Ipomeas patatas* (sweet patato) as a multiple stress responsible gene also called (*MuSI*) encoding a 27 kDa protein [36]. In addition, a hypothetical REF-like protein from *Salaginella moellendorffii* (a lycophyte from an ancient vascular plant lineage) was identified and could imply an old evolution of this plant family. Amazingly, another hypothetic partial protein from *Amblyomma maculatum* (a specie of tick) appeared in the search but is probably not connected and remains to be fully identified. The full alignment of the REF/SRPP proteins from *H. brasiliensis* is showed in Supplementary Figure 1, and the homology of both REF (P15252) and SRPP (O82803) genes we used in this study is presented in Figure 2A with the sequence consensus of the hevea family. This important homology (>40%) was previously described [8], [13], [29], [37] and their possible role during stress was previously proposed as their mRNA expression increases upon tapping [32], [33].

Additionally, we note here that SRPP clearly differs from REF by a long additionnal C-terminal part. The function of these proteins is still to determine, but surely they are the two major proteins present in *Hevea brasiliensis* latex as shown in Figure 2B. In order to have a better understanding of their role in latex, we synthesized corresponding coding sequences optimized for *E. coli* expression and purified the recombinant histidine-tagged proteins to homogeneity.

### REF Displays Aggregation Properties

One of the first observation we made working with REF and SRPP, was a spontaneous aggregation of REF in physiological conditions (PBS). As a slight auto-assembling of SRPP was previously reported [8], then we tried to incubate both proteins at 20 µM in PBS, 37°C for 3 h. We observed an instantaneous precipitation of REF whereas SRPP never showed such behavior (Figure 2C). Indeed through all our study SRPP displayed a quite good stability and solubility. Physico-chemical properties of REF and SRPP are presented in Supplementary Table S1. We note that the net charge of SRPP (−6) differs from that of REF (−2) and could take part in the relative stability of SRPP. In addition, hydropathy plots by the Kyte and Doolittle method [38] show two hydrophobic proteins (Supplementary Figure S2), with REF having a negative score (−0.084; Supplementary Table S1) and a peak >2 in the window 90–100. From a rapid secondary structure prediction analysis (PredictProtein, https://www.predictprotein.org, [39]), we expected that both proteins could contain more than 60% of α-helices and also possess a potential transmembrane domain in their N-terminal part. But obviously REF was prone to aggregation.

We tested beta-aggregation propensity by the Tango method (Supplementary Figure S3) [40]. REF presents two important peaks (up to >95%) for aggregation (amino acids around 25–50 and 85–100), whereas two smaller peaks (>70%) are observed with SRPP (amino acid around 25–50 and below 150). The aggregation window around amino acid 150 is only present in SRPP, but the aggregation window 90–100 in REF seems critical for aggregation. We note in SRPP a GVV motif in position 92 that could play the role of beta-sheet breaker.

As aggregation could be the sign of amyloidogenesis, and that amyloid may show a certain resistance to proteases, we compared the degradation of both proteins by proteinase K at 37°C (PK). In Figure 2D, REF aggregates demonstrate quite a good resistance to PK with a resistant main core around 13–14 kDa. SRPP digestion contrarily leads to 2 resistant fragments of 13–14 kDa. We also noted during the course of this experiment, the apparition of higher bands in both protein samples, which could be also attributed to concomitant auto-assembling. This could confirm that SRPP has also potential auto-assembling properties.

We pursued the analysis of these aggregative properties using specific amyloid dyes. REF aggregates are clearly stained by Congo red, with a classical red shifted spectrum and also show birefringence under cross-polarized light, which is typical of amyloids (Figure 3A–B). Only a weak binding of CR was observed with SRPP but not correlated with any visible aggregations. This could still be related to the formation of very small assemblies of SRPP. Both proteins showed Thioflavine T (ThT) binding, with an increase of ThT fluorescence upon binding (Figure 3C). Interestingly, both proteins strongly bind to Bis-ANS, which is an amphipathic dye widely used to characterize partially folded intermediates and proteins of hydrophobic nature (Figure 3D). If REF shows aggregation and dye-binding characteristics of amyloids, it shares also with SRPP a common hydrophobic nature, which could take part in their interactions with membranes of rubber particles.

### REF Exhibits an Amyloid Signature

Proteins used for the following structural study were proteins incubated at 20 µM, pH 7.4, 37°C for 3 h. Then we first performed circular dichroism. The CD spectra of soluble SRPP shows as expected from the prediction a pattern rich of α-helices with a classical double negative peak around 205–215 nm, whereas aggregated REF shows a different spectra with a weaker negative peak at around 217 nm, which could be more characteristic of a β-sheet content (Figure 4A).

To go further into their respective secondary structural composition we used ATR-FTIR spectroscopy and analyzed the results according to our previous observations (aggregation, helix, hydrophobicity). Figure 4B shows the ATR-spectra in the range 1780–1450 cm^−1^, characteristic of the amide I and II vibrations of the peptidic bands (CO-NH). Analyses of the amide I band and deconvolutions were performed as described in Materials and Methods. The amide I recorded was deconvoluted into six main structures: amide group in hydrophobic domain (1685 cm^−1^), β-turns (1670 cm^−1^), random coils (1656–57 cm^−1^), α-helices (1642–1646 cm^−1^), β-sheets (1629–1630 cm^−1^), and finally aggregation (1614 cm^−1^). The components of each band and the percentage of each structure are presented in Table 1. This result confirms the high content of β-sheets in REF aggregates, whereas SRPP contains more helices and random coils. As we observed the REF aggregates by TEM (Figure 4C), we noticed that the full sample was highly aggregated in a seemingly amyloid “amorphous” state. It is known that highly hydrophobic sequences may have a strong tendency to form amorphous cross-beta aggregates [41]. The presence of cross-β structures was confirmed by a X-ray diffraction (WAXS) analysis, which showed for REF the two classical reflexions of amyloids around 4.7 and 10 Å (Figure 4D). As the aggregation of REF was obviously so fast and intense after 3 h at 37°C, that we could not observe any amyloid fibers in the sample, we analyzed samples of REF prepared at 20°C for 20 min to reduce the amyloidogenesis process. In these conditions, we were able to visualize by TEM REF amyloid fibers (Figure 5). They were micrometer-long fibrils, quite large (usually >10 nm in diameter), unbranched and assembling laterally. A lot of more complex structures related to amorphous amyloids were also observed.

We are therefore in presence of two closely related proteins, which display completely different structural features. Indeed, REF shows all the characteristic of an amyloid protein with high aggregation properties. To our knowledge, this is the first report of an amyloid protein observed in the plant kingdom.

### REF and SRPP Interact Differently with Membrane

As REF and SRPP proteins have been previously visualized by immunogold labeling TEM, bound to the surface of Large Rubber Particles (LRP) and Small Rubber Particles (SRP) [15], [17], we investigated their interactions with model lipid membranes. The rubber particles are surrounded by a biomembrane organized as a monolayer and constituted of proteins, phospholipids (mainly phosphatidyl choline and ethanolamine), glycolipids and β-sitosterol [19], [42], [43]. First we studied the direct interaction with bulk lipids blotted on PVDF membranes, to compare the difference of interactions with various kinds of lipids. We chose to use asolectin, DMPC, and cholesterol. Asolectin is a complex mixture of lipids derived from soybean membranes, which is composed of many lipids found in latex, and PC is also highly present in rubber particle membrane [44]. From lipid dot blots presented in Figure 6A we can clearly visualized that both REF and SRPP strongly interacted with asolectin and cholesterol, but weakly with DMPC. In the experiment, the lipids in bulk were presented on the blot in a non-oriented fashion. Both polar heads and lipid tails could be recognized by the proteins.

To go deeper in our characterization of the interaction with membranes, we used then lipid monolayers as model. The zwitterionic DMPC or asolectin monomembranes are realized at the air-water interface and the protein is injected in the aqueous subphase (Figure 6B). The interaction is visualized by surface pressure (Table 2 and Table 3) and ellipsometry. Our first astonishing observation was that both proteins were extremely reactive alone at the air-water interface. Compared to a previous Langmuir study we performed on HET-s(218–289) amyloids [45], REF and SRPP were forming very rapidly (less than 30 min) a thick homogeneous protein film at the air/water interface (REF ∼136 Å and SRPP ∼36 Å). The surface pressures for both proteins alone at the interface was also quite strong (REF ∼24 mN/m and SRPP ∼28 mN/m). Apparently, this effect was stronger for REF, but is consistent with the fact that they are both highly hydrophobic and confirmed the high binding to Bis-ANS and hydropathicity (Figure 3D; Supplementary Table 1, Supplementary Fig. 2). When we added the proteins under the DMPC and asolectin monolayers the effect was different. For REF in both cases, the protein came rapidly to the contact of the lipid polar heads and clearly modified the lipid monolayer (Figure 6B). The interaction was stronger with DMPC (Table 3), but each lipid presented a relatively high increase of the interface thickness (∼66 Å and ∼63 Å) and morphology, which is the observation of a massive insertion into the monolayer as revealed by the increase of the surface pressure. In the case of SRPP, the proteins come rapidly to the contact of the lipid polar heads, slightly thickening the interface film (∼26 Å) below the lipids but not modifying the lipid film morphology. It was previously observed that SRPP could be released (and REF to a lower extend) from RP into the cytosol upon osmotic lysis [33]. This confirms also a weaker interaction of SRPP compared to REF towards membranes.

In conclusion, both proteins are very surface active, but display different behaviors towards membrane monolayers. In Figure 7, we proposed and schematized a model of the interactions of SRPP as bound to the surface of SRP and REF as deeply inserted into LRP. Two models of interaction which could lead to different functions.

## Discussion

In this study, we uncovered that two related proteins REF and SRPP present in hevea latex and sharing sequence homology, present clearly different structures. Our results show that at least *in vitro* in physiological condition (pH 7.4), SRPP is an α-helical protein whereas REF is an amyloid. The pH of cytosol latex is usually neutral (6.9±0.4) but it also exists a direct relationship between slightly alkaline pH after tapping and increased rubber production [46]. So far amyloid proteins have never been characterized in latex, but various proteinic microhelices and microfibrils have been visualized in lutoids, which are a kind of lysosomic vacuoles present in latex [2], [47], [48]. Some of them were referred as “vegetative storage protein” [49].

In the last decade numerous amyloid fibers with functional activities have been identified in bacteria, fungi, insects, invertebrates and humans (for review see [50]). They fulfill crucial functions as for example adhesion to surface, cell aggregation, biofilm formation, molecular sequestration and storage [51]. Many amyloids are now recognized as important nanobiomaterials [52]. For example, Curli an amyloid produced extracellularly by many *Enterobacteriaceae*, generates a matrix for surface adhesion and interactions with other bacteria, conferring a colonizing advantage [53], [54]. Mod5, a tRNA isopentenyl transferase is a yeast prion which regulates acquired drug resistance and cellular adaptation to environmental stress [55]. Other amyloids called hydrophobins are small (about 100 amino acids) highly hydrophobic proteins produced by filamentous fungi, very surface active (as REF and SRPP) and able to form robust polymeric monolayers with coating properties [56], [57]. In latex, both proteins are really tighly bound to SRP and LRP, and their extraction from rubber particles requires strong treatments with detergents [12], [33]. From early studies, large rubber particles were thought to be completely coated by a “proteinaceous film” of REF, possibly in more than one layer [12], [58]. From our ellipsometric study, it seems also from the thickness and pressure that several layers of REF could be present through the monomembrane, but it would now be interesting to determine the structure of the inserted REF. Is it the amyloid form or amphipathic α-helices anchored and stabilized by the lipids? In fact, it is believed that many, if not all proteins are able to undergo this structural transition into amyloid when under the appropriate conditions [59], [60]. In addition, temperature, pH, surfaces and membrane composition clearly appear as key factors in triggering folding [61], [62], [63].

In the case of SRPP, we did not observed an amyloid state or aggregates, but SRPP from latex was also suspected of aggregation [8], [15]. In addition SRPP fragmentation was previously reported [8], [15] and it may occur at specific sites on the molecule [8], which could be corresponding to REF amyloid core as we confirmed by proteinase K treatment. The extra C-terminal part present on SRPP and the negative charge (−6) may therefore account for the relative stability of this protein. In addition, as a glycoprotein SRPP is also believed to contribute to latex coagulation by an agglutination mechanism [64], and rubber synthesis has been shown to be inhibited by hevein, a lectin present in lutoids and able to interact with SRPP [25].

Therefore, if REF and SRPP take part in the rubber biosynthesis process, we may suspect their intervention in the coagulation of rubber particles *via* mechanisms of agglutination or aggregation more than *via* an enzymatic activity. But it is not excluded that they also could participate in the recruitment of rubber enzymes to the surface of latex globules.

## Supporting Information

Figure S1 **Sequence homology of REF/SRPP/SRP family in** ***Hevea brasiliensis*** **.** Various proteins from *H. brasiliensis* were identified by Blast 2.2.18 (http://www.phylogeny.fr). The 7 sequences with assigned Genbank or Swiss-Prot accession numbers (left) were aligned using ClustalW (http://www.genome.jp/tools/clustalw/program) and shaded by using GeneDoc 2.7.0 (http://www.nrbc.org/downloads). The consensus is presented below amino-acid sequences. Black shadings highlights 100% homology, gray shadings show only partial consensus >50%. Proteins used in this study are REF P15252 (top) and SRPP O82803 (bottom).
(TIF)

Figure S2 **Kyte-Doolittle hydropathy plots** of REF (**A**) and SRPP (**B**) obtained at http://gcat.davidson.edu/rakarnik/kyte-doolittle.htm. Window size 9 was applied.
(TIF)

Figure S3 **TANGO plots** of REF (**A**) and SRPP (**B**) obtained at http://tango.crg.es/. Beta aggregation plots at presented at pH 7.0 and 25°C.
(TIF)

Table S1 Physico-chemical parameters of His-tagged REF and SRPP.
(DOCX)
