# Supplementary figures and images for: Rubber Elongation Factor (REF), a Major Allergen Component in Hevea brasiliensis Latex Has Amyloid Properties
Source: PLoS One. 2012 Oct 25;7(10):e48065. doi: 10.1371/journal.pone.0048065 (PMC3485013; doi:10.1371/journal.pone.0048065)

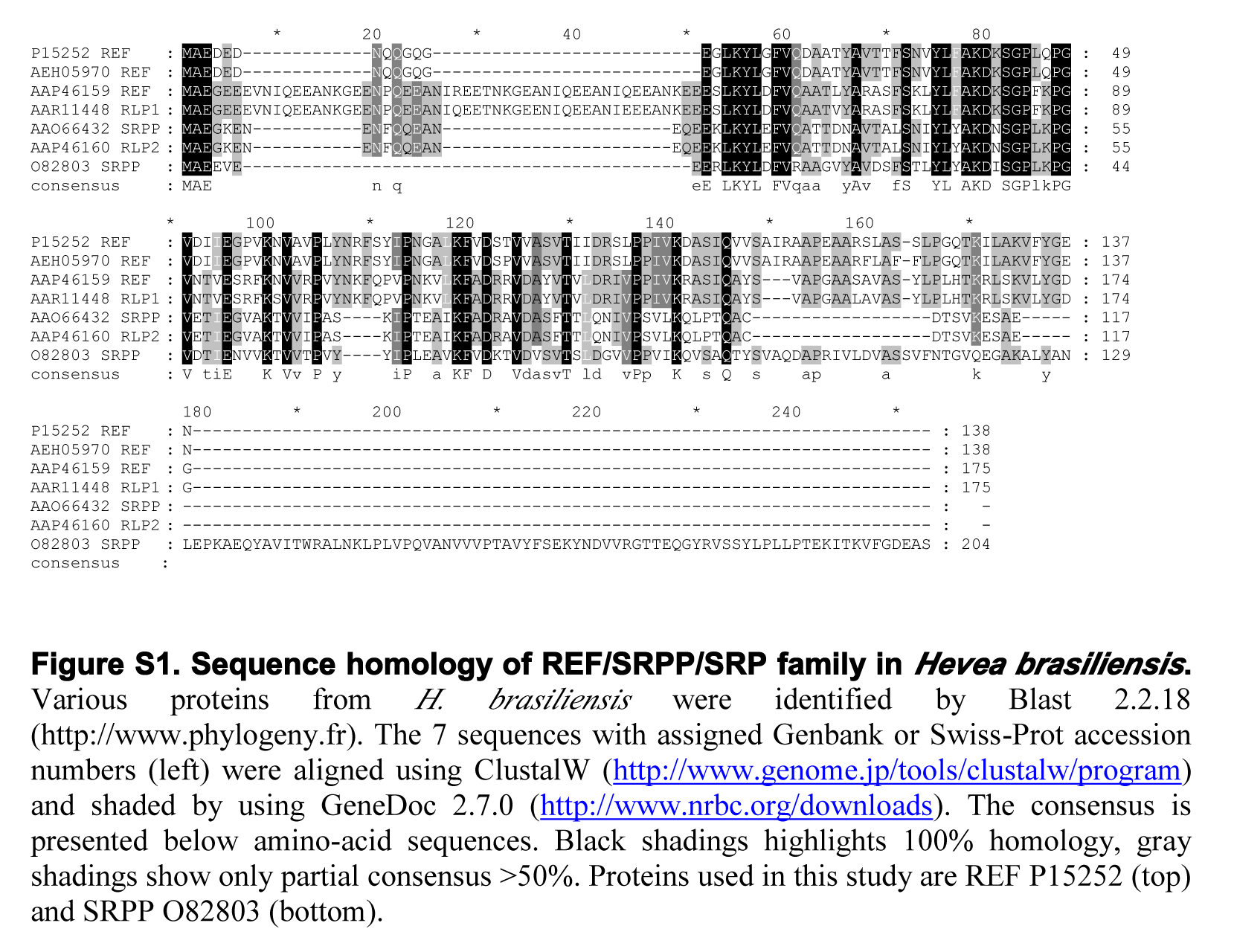

Supplement: Figure S1 — Sequence homology of REF/SRPP/SRP family in Hevea brasiliensis . Various proteins from H. brasiliensis were identified by Blast 2.2.18 (http://www.phylogeny.fr). The 7 sequences with assigned Genbank or Swiss-Prot accession numbers (left) were aligned using ClustalW (http://www.genome.jp/tools/clustalw/program) and shaded by using GeneDoc 2.7.0 (http://www.nrbc.org/downloads). The consensus is presented below amino-acid sequences. Black shadings highlights 100% homology, gray shadings show only partial consensus >50%. Proteins used in this study are REF P15252 (top) and SRPP O82803 (bottom). (TIF) [file pone.0048065.s001.tif]

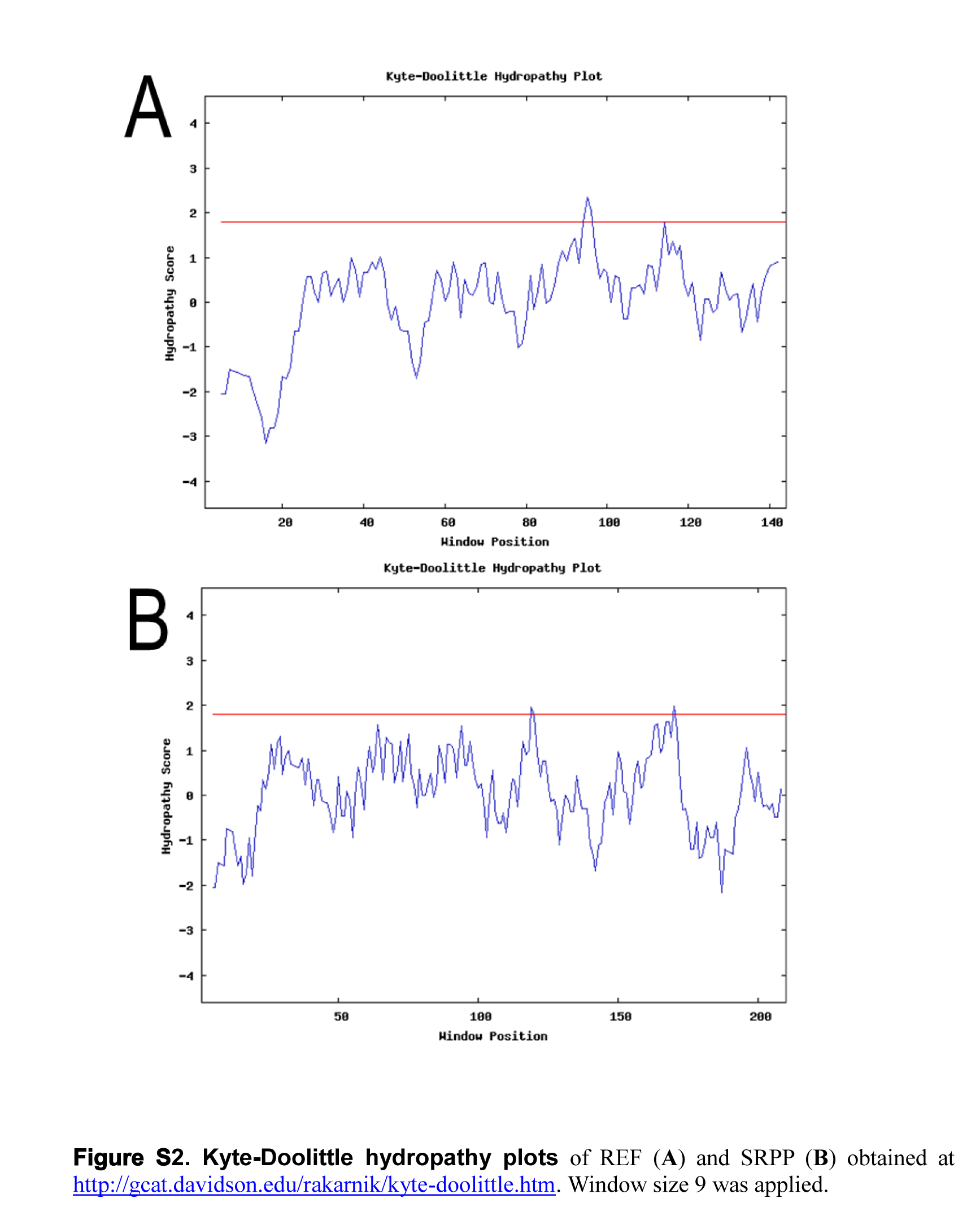

Supplement: Figure S2 — Kyte-Doolittle hydropathy plots of REF (A) and SRPP (B) obtained at http://gcat.davidson.edu/rakarnik/kyte-doolittle.htm. Window size 9 was applied. (TIF) [file pone.0048065.s002.tif]

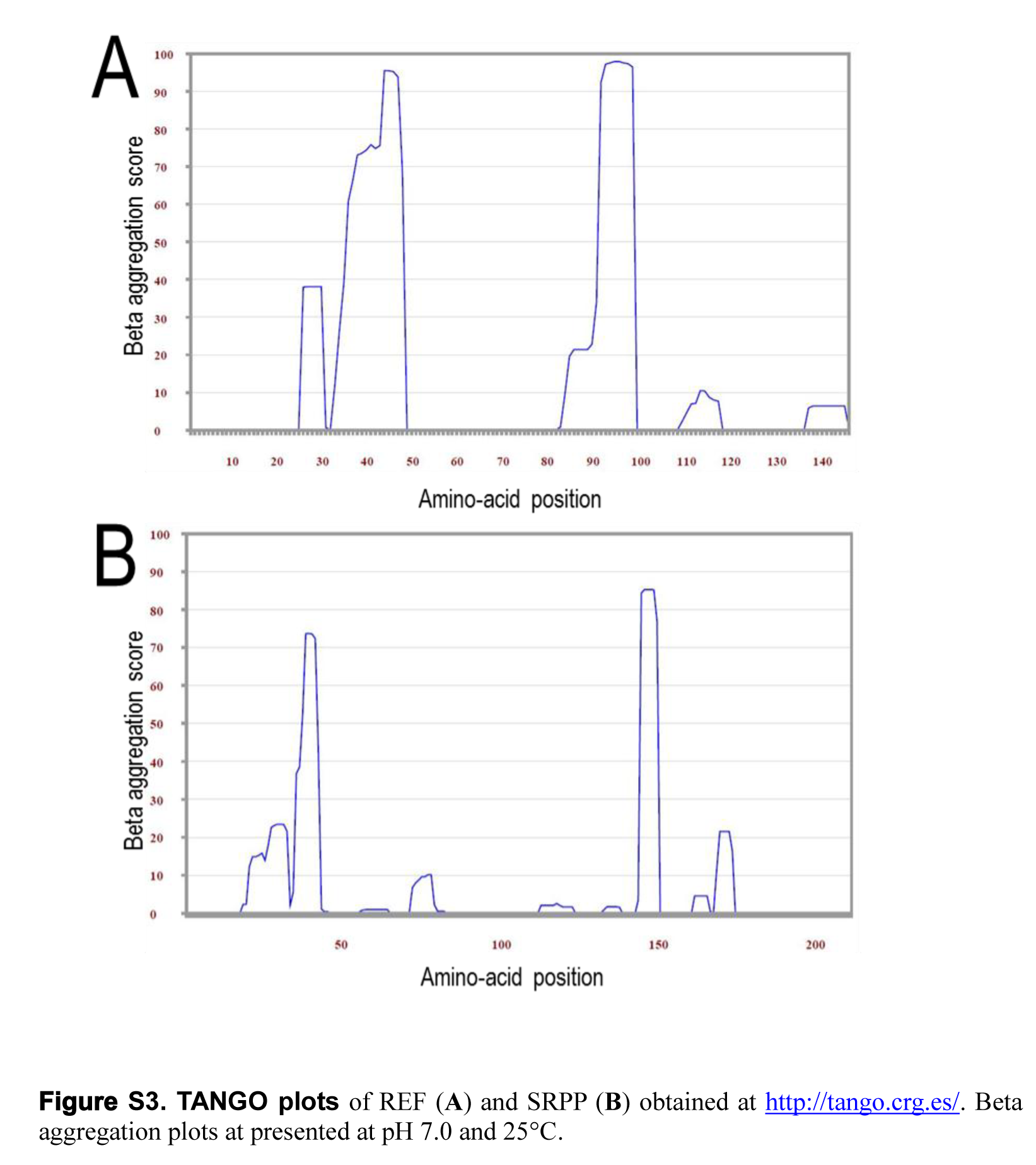

Supplement: Figure S3 — TANGO plots of REF (A) and SRPP (B) obtained at http://tango.crg.es/. Beta aggregation plots at presented at pH 7.0 and 25°C. (TIF) [file pone.0048065.s003.tif]
