# Supplementary material for: Rubber Elongation Factor (REF), a Major Allergen Component in Hevea brasiliensis Latex Has Amyloid Properties
Source: PLoS One. 2012 Oct 25;7(10):e48065. doi: 10.1371/journal.pone.0048065 (PMC3485013; doi:10.1371/journal.pone.0048065)
Supplement: Table S1 — Physico-chemical parameters of His-tagged REF and SRPP. (DOCX) [file pone.0048065.s004.docx]

**Table S1.** Physico-chemical parameters of His-tagged REF and SRPP proteins.

| *proteins* | *amino acids* | *MW* | *pI* | *ext. coefficient* | *net charge* | *hydropathicity* |
| --- | --- | --- | --- | --- | --- | --- |
|  |  | ***(Kda)*** |  | ***(L.mol^-1^.cm^-1^)*** |  |  |
| REF | 147 | 15.8 | 6.3 | 8940 | -2 | -0.084 |
| SRPP | 213 | 23.4 | 5.5 | 24870 | -6 | 0.003 |

Data at pH 7.4, and grand average of hydropathicity (GRAVY) were estimated from the amino-acid sequence of each protein fused to the N-terminal His tag (sequence MHHHHHHGS). Data were obtained from ExPASy proteomics Server, htttp://expasy.org/tools/protparam.html.
